# Management of a Traumatic Multiligament Knee Injury With Coinciding Patellar Tendon Rupture: A Case Report

**DOI:** 10.1155/cro/2859359

**Published:** 2026-02-23

**Authors:** Nicholas I. Chiaramonti, Nathan M. Krebs

**Affiliations:** ^1^ College of Medicine, Central Michigan University, Mount Pleasant, Michigan, USA, cmich.edu; ^2^ Department of Orthopedics and Sports Medicine, MyMichigan Medical Center Saginaw, Saginaw, Michigan, USA

**Keywords:** case report, multiligament knee injury, patellar tendon rupture

## Abstract

Although ligamentous injuries to the knee are a common occurrence, concomitant rupture of the patellar tendon (PT) is a rare finding. We present the case of a 28‐year‐old male who sustained a multiligamentous injury of the knee, which involved the anterior cruciate ligament (ACL), posterior cruciate ligament (PCL), and medial collateral ligament (MCL) along with a rupture of the PT secondary to a dirt bike accident. We also review the current treatment guidelines for multiligamentous knee injuries based on the available literature. This unique presentation was treated surgically using a two‐staged approach: an initial acute repair of the PT and MCL, followed by an arthroscopic reconstruction of the ACL and PCL using allograft tissues. This approach, combined with an aggressive rehabilitation program, can lead to a satisfactory clinical outcome for a multiligamentous knee injury in combination with a rupture of the PT.

## 1. Introduction

Injuries to any one of the knee ligaments—including the medial collateral ligament (MCL), lateral collateral ligament (LCL), anterior cruciate ligament (ACL), or posterior cruciate ligament (PCL)—in isolation are commonly seen in the United States, affecting approximately 400,000 individuals each year [1]. Less common, though still routinely seen in orthopedic and sports medicine clinics, are multiligamentous knee injuries—those that simultaneously affect two or more of the aforementioned ligaments. Woo et al. estimate only 20,000 multiligamentous knee injuries in the United States each year [2]. Furthermore, a rupture of the patellar tendon (PT) or quadriceps tendon is seen in a mere 0.19% of all knee injuries [3]. Here, we present a case of an extremely rare injury pattern: a multiligamentous knee injury with concomitant rupture of the PT. In a 2019 review, Quinn et al. identified only 21 reported cases of multiligament knee injury with a simultaneous PT rupture in the literature [4]. Interestingly, all 21 cases reported combined ACL/MCL injury in addition to the PT rupture [4].

Here, we present a unique case of a simultaneous ACL/PCL/MCL injury with a concomitant rupture of the PT. We also report the clinical findings and operative management using the most current techniques. To our knowledge, this is the first reported case of this specific injury pattern.

## 2. Case Presentation

### 2.1. Preoperative Assessment

A 28‐year‐old male presented to the emergency department shortly after a severe dirt bike accident. The patient was traveling at 25 mph on a dirt bike when he lost control and crashed, sustaining a severe right knee injury. His past medical history, family history, and psychosocial history were assessed and are noncontributory to this presentation. In addition to assessing cognitive function and activating trauma protocol, the emergency department consulted an orthopedic surgery to evaluate the right knee.

On initial physical exam, there was an obvious deformity of the knee, as the patient held the extremity in a hyper‐flexed position. Strong pulses were noted distal to the knee, which lowered suspicion of vascular damage. The patient was sedated by the ED provider for emergent closed reduction of the knee by applying longitudinal traction to the extremity and appreciating an audible clunk as the joint was reduced. A knee immobilizer was then applied to the right lower extremity. The patient remained neurovascularly intact following the reduction. Initial x‐ray of the region revealed patella alta (high‐riding patella) but was negative for acute fracture. CT scan of the knee was ordered by the emergency department demonstrated hypodensity within the PT substance suggestive of inflammation around the PT with concern for injury. CT scans of the head, spine, and chest revealed no abnormalities. The patient was otherwise stable. Due to the vascular risk associated with knee dislocations, there was a prolonged concern for his vascular status. Therefore, he remained in the emergency department for a 24‐h observation period with low‐molecular weight heparin (LMWH) for deep‐vein thrombosis (DVT) prophylaxis and regular compartment checks of the right lower extremity to monitor for compartment syndrome. He was then discharged from the ED with LMWH 40 mg subcutaneously twice daily for DVT prophylaxis and plans for MRI of the knee in the outpatient setting.

Magnetic resonance imaging (MRI) of this knee was obtained 5 days after the initial injury after being further evaluated in clinic. As shown in Figure 1, the MRI confirmed the following: (1) a complete tear/rupture of the PT at its midsubstance, between the patellar and tibial attachments, (2) a high‐grade, near complete tears of the ACL and PCL, and (3) a complete tear of the superficial MCL at the level of the joint line, along with a complete tear of the meniscofemoral component of the deep MCL. With these findings, an operative plan was developed in two stages: first, an immediate repair of the PT and MCL; second, a subsequent arthroscopic reconstruction of the ACL and PCL.

Figure 1MRI findings (a) sagittal section of the right knee revealing a rupture of the patellar tendon at its midsubstance, (b) sagittal section of the right knee revealing high‐grade, near complete tears of the ACL and PCL, and (c) coronal section of the right knee revealing a complete tear of the superficial MCL at the level of the joint line.

**Figure:**
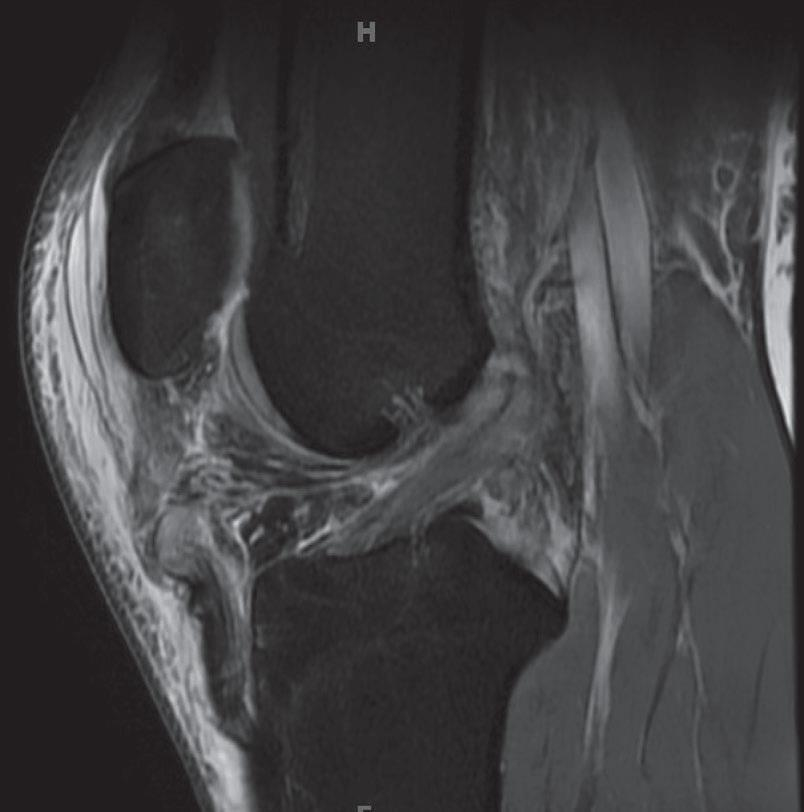
(a)

**Figure:**
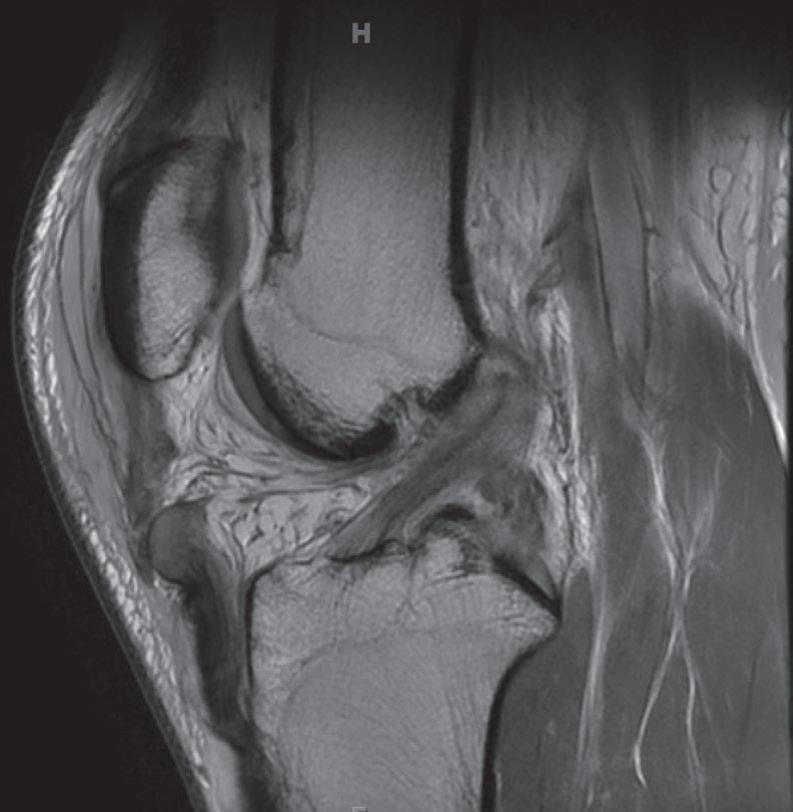
(b)

**Figure:**
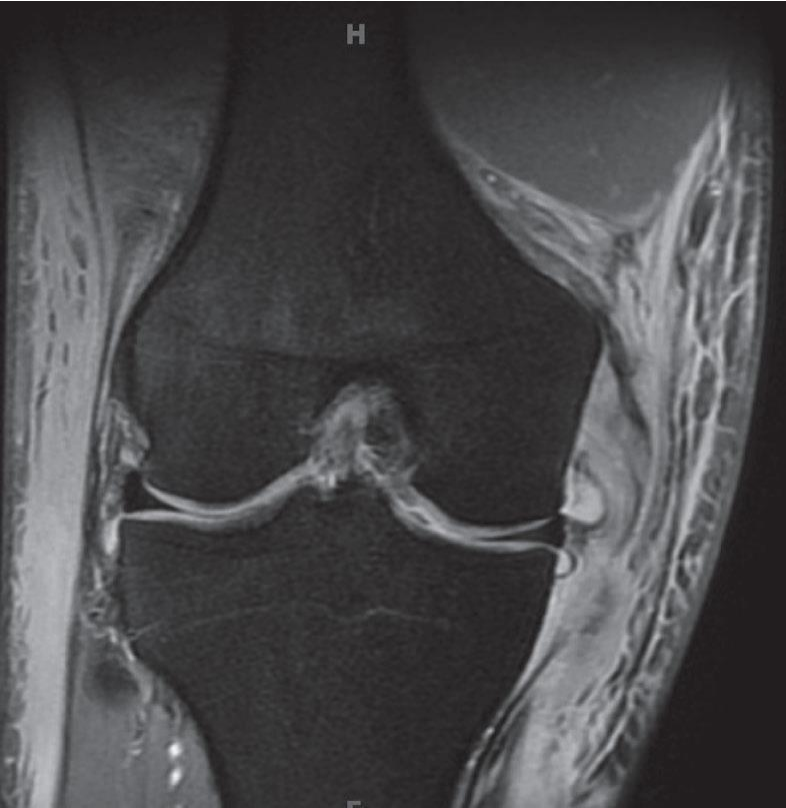
(c)

### 2.2. Surgery #1: PT and MCL Repair

Prior to the PT and MCL repair, physical exam of the right knee under anesthesia again revealed Grade 3+ instability [5] to valgus stress between 0° and 30°, positive Lachman exam, and positive anterior and posterior drawer tests. There was no evidence of posterolateral corner instability. The right knee was then prepped and draped using a sterile technique, and the tourniquet was inflated.

An anterior midline surgical approach was used to access the patellar paratenon, which was opened to reveal complete disruption of the medial and lateral retinaculum and complete rupture of the midsubstance of the PT (Figure 2a). This area was thoroughly irrigated and debrided of nonviable tissue, and repair was initiated using two locking Krakow stitches on each tendon edge using nonabsorbable suture. The inferior border of the patella was then exposed, and biocomposite swivel lock anchors were placed to utilize the internal brace technique to augment the repair and allow early mobilization of the knee [6]. The knee was kept at roughly neutral extension while the central core strands from the Krakow stitches were tied to repair the PT. The knee was flexed to approximately 30°, whereas the preloaded internal brace sutures were crossed from each anchor and fixated along the medial–lateral aspect of the tibial tubercle. Importantly, the knee was able to be ranged from 0° to 90° at this time, displaying no evidence of overtensioning. The medial and lateral retinaculum were then repaired. This finalized the repair of the PT (Figure 2b), and the wound was closed.

Figure 2Intraoperative images for Surgery #1 (a) anterior view of the right knee prior to revealing a rupture of the patellar tendon at its midsubstance, (b) anterior view of the right knee after surgical repair of patellar tendon, (c) anteromedial view of the right knee revealing a complete tear of the superficial MCL at the level of the joint line, and (d) anteromedial view of the right knee after surgical repair of the MCL.

**Figure:**
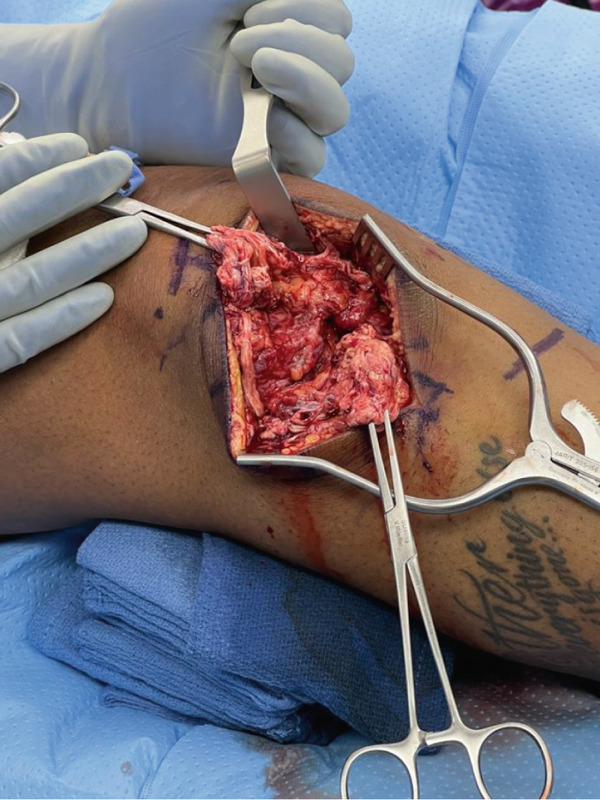
(a)

**Figure:**
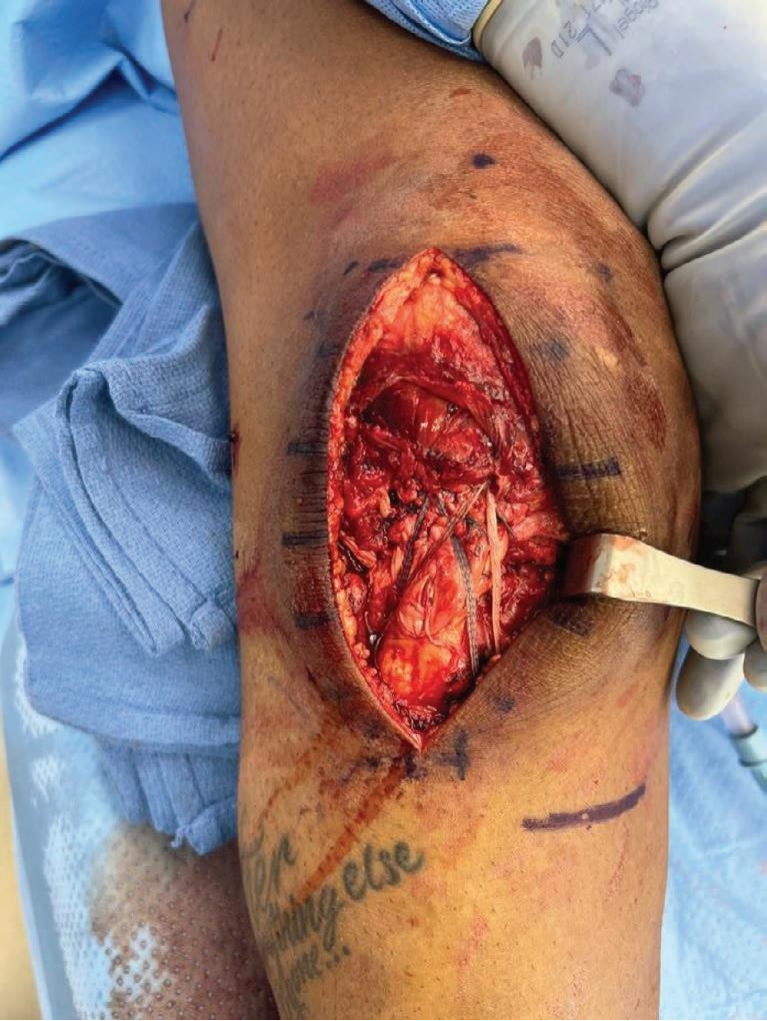
(b)

**Figure:**
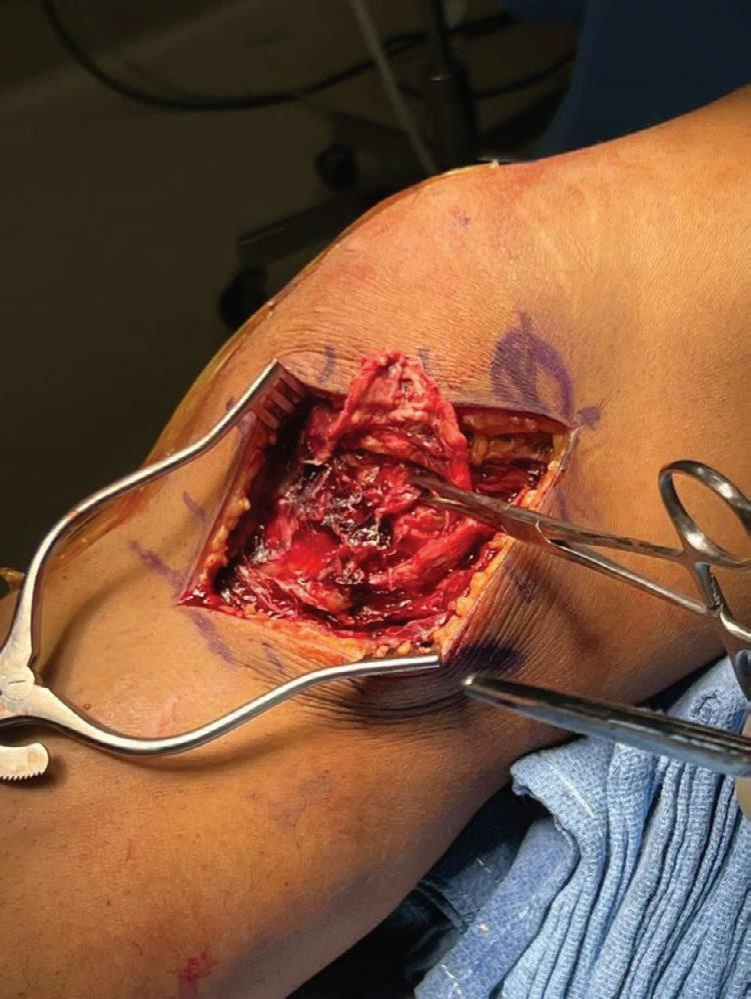
(c)

**Figure:**
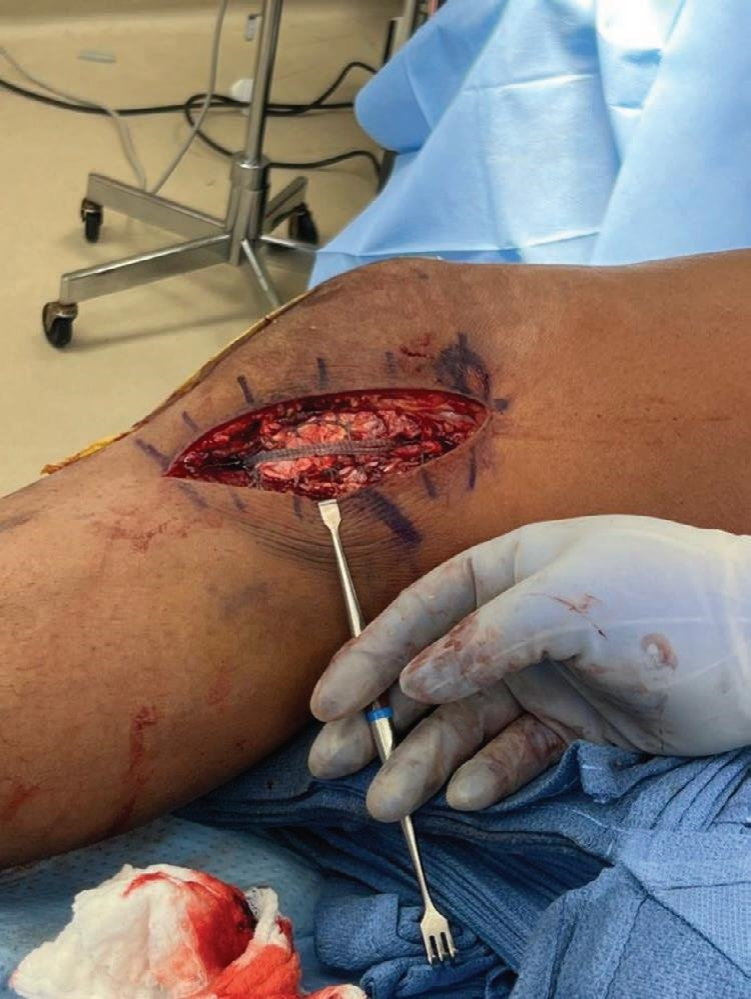
(d)

For the next stage of the first surgery, our incision started at the medial condyle and proceeded 6 cm distally to the joint line at the native insertion of the MCL. Dissection was carried down through the sartorial fascia. This revealed a complete disruption of the superficial and deep MCL at the level of the joint line extending deep to the capsule and periosteum (Figure 2c). The deep MCL was repaired back down to the periosteum using a suture anchor. The proximal segment of the MCL was repaired to its native insertion at the medial epicondyle using a suture anchor. Finally, the more posterior MCL tissue was imbricated to the posterior border of the repaired MCL to further restore stability to the posterior medial corner. The repairs were performed as the leg was held at 30° flexion, neutral rotation, and with a slight varus stress applied to the knee. This finalized the repair of the MCL (Figure 2d), and the wound was closed.

Upon conclusion of the first surgery, the patient was placed in a total range of motion (TROM) brace locked in extension and kept 25% partial weight‐bearing on the right lower extremity. Physical therapy was initiated at the first postoperative visit 2 weeks after the surgery to begin passive ROM exercise with the goal of 0°–90° of motion by 6 weeks postop.

### 2.3. Surgery #2: ACL and PCL Reconstruction

About 8 weeks after the MCL and PT repair, the patient underwent the second stage of surgical repair: arthroscopic reconstruction of the ACL and PCL. Preoperative examination under anesthesia revealed a much improved stability with valgus stress testing, demonstrating healing of the prior MCL repair. Range of motion achieved was full extension to 85° of flexion, possibly due to some arthrofibrosis that developed between surgeries. Anterior and posterior drawer tests were positive. The right knee was then prepped and draped using a sterile technique, and the tourniquet was inflated.

The standard arthroscopic portals were initially established, then a posteromedial portal was later established to assist with the PCL reconstruction. Although scar tissue was removed to allow adequate exposure of the joint space, the ACL and PCL allografts were prepared, placed under tension, and wrapped in vancomycin‐soaked gauze. The ACL allograft measured 10 mm in diameter by 68 mm in length. The PCL allograft measured 11 mm in diameter by 90 mm in length. Guide selection and identification of visual landmarks are important prior to proceeding with tunnel creation. The femoral ACL tunnel placement was identified using the lateral intercondylar ridge and bifurcate ridge as primary landmarks. The tunnel was placed low and posterior on the lateral femoral condyle. The tibial ACL tunnel was created using the medial tibial spine and anterior root of the lateral meniscus as visual landmarks. A tibial tunnel drilling guide was set at 55° to target the native tibial footprint, with care taken to avoid injury to the anterior root of the lateral meniscus. As for the PCL tunnel creation, the femoral tunnel was created using the medial femoral condyle and articular cartilage margin as visual landmarks. The tunnel was placed on the lateral aspect of the medial condyle, just proximal to the cartilage line. The tibial PCL tunnel was placed over the posterior flat spot of the tibia, slightly lateral to the midline. This was accomplished using the PCL facet and champagne‐glass drop‐off as visual landmarks, with special care taken to avoid popliteal artery injury.

The all‐inside technique [7] was utilized for both the PCL and ACL reconstruction. PCL tunnels were created via 25 mm of retrograde drilling through the posterior tibia and the medial condyle of the femur. ACL tunnels were then created via 25 mm of retrograde drilling through the lateral femoral condyle and through the native tibial footprint. The prepared allografts were passed through the tunnels, first the PCL, then the ACL. Fixation was provided by femoral and tibial cortical buttons. Each graft was tensioned appropriately after cycling the knee 30 times (Figure 3).

Figure 3Postoperative x‐ray findings (a) AP and (b) lateral views of the knee demonstrating cortical button fixation for ACL and PCL reconstruction. These x‐rays were obtained 1‐week postoperatively.

**Figure:**
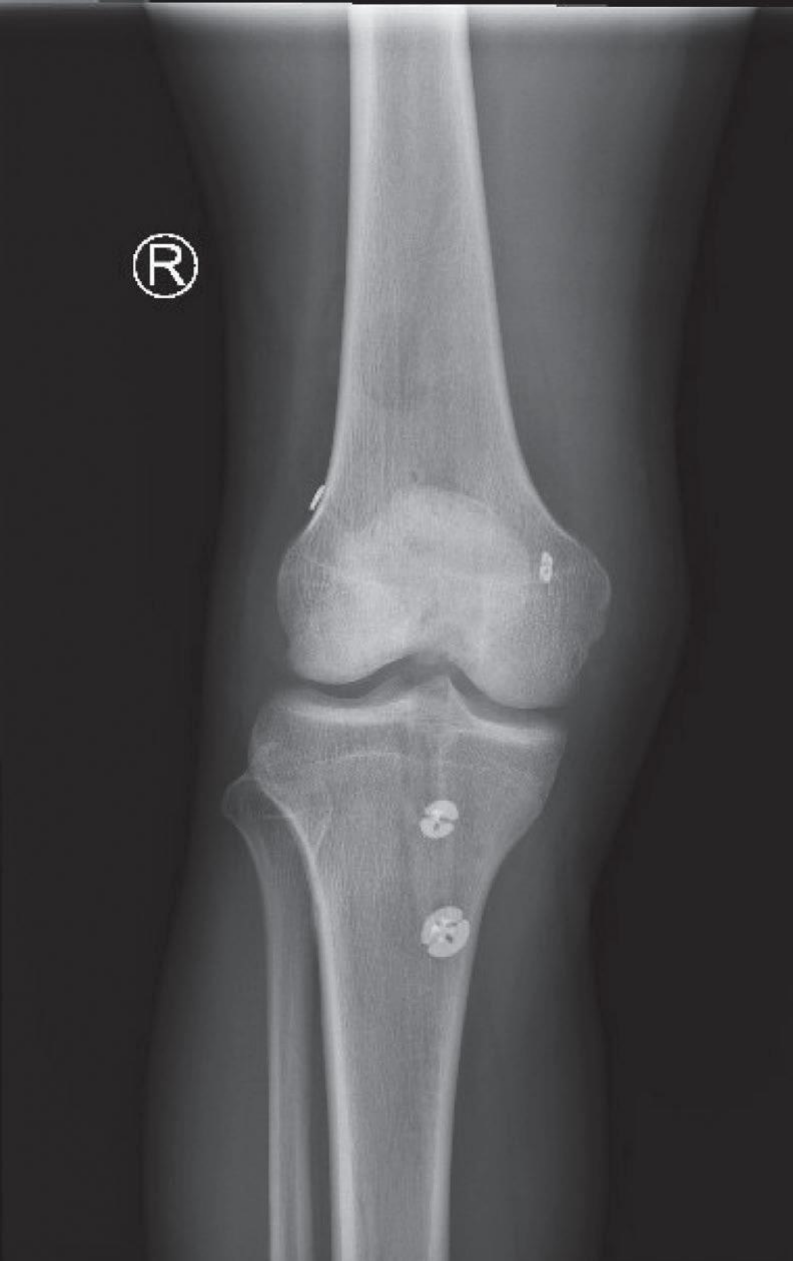
(a)

**Figure:**
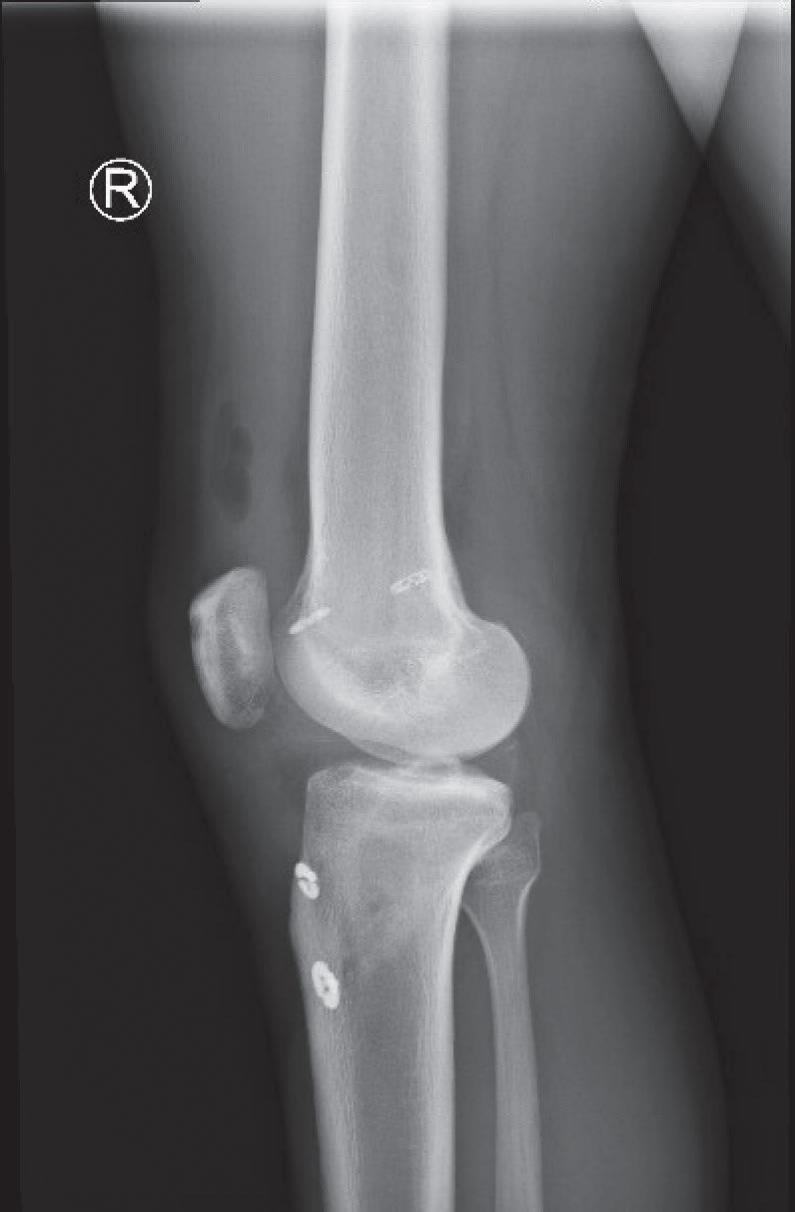
(b)

At the conclusion of this surgery, the knee was able to be ranged from full extension to 90° flexion, and there was stability to the anterior and posterior drawer tests. A TROM brace was applied and locked in extension, and the patient was advised to be partial weight‐bearing with crutches.

### 2.4. Postoperative Management and Follow‐Up

The postoperative management for this patient was focused on returning the patient to full range of motion while still allowing graft healing. Thus, physical therapy was tasked with aggressively working on range of motion. One week after the operation, the patient displayed full extension but flexion to only 30°. At 3 weeks postop, the recovery was progressing well, but flexion was still limited to 55°. At 6 weeks postop, flexion remained limited at approximately 70°, despite aggressive physical therapy of three times per week and the use of oral steroids.

Due to a minimal improvement in the range of flexion at the 13‐week pos‐op visit, we planned a surgical manipulation under anesthesia and arthroscopic lysis of adhesions. During this procedure, a dense scar tissue was removed from the entire anterior interval and the medial and lateral gutters. Extensive adhesions were also released from the suprapatellar pouch. Following the adhesiolysis, the patient was able to be ranged from 0° extension to 135° flexion while under anesthesia. Physical therapy was continued after the procedure.

At follow‐up of 6 months after the initial injury, the patient was healing very well and achieved range of motion from full extension to 120° of flexion. At the 8‐month follow‐up, flexion improved to 130°. By the 1‐year follow‐up appointment, the patient was cleared to return to all normal activities. The traumatic injury to the knee was well‐healed and self‐reported satisfaction with the treatment was very high. At his 2‐year final follow‐up visit, the patient continued to report great satisfaction and no concerns with the knee.

## 3. Discussion

Rupture of the PT is an exceedingly rare injury. It is even less common to see this finding in the presence of a coinciding ligamentous knee injury. To the best of our knowledge, there have been only 33 reported cases of this injury pattern (Table 1). Many of these cases report injury to only a single ligament, in addition to the PT rupture. Of the 22 cases reporting an injury to multiple ligaments in the knee, they all find a combined ACL/MCL tear alongside the PT rupture. Therefore, we report the first published case of an ACL/MCL/PCL ligament tear in addition to a PT rupture.

**Table 1:** Documented cases of combined PT rupture and ACL +/− MCL injury.

Study	Year of publication	Case	Injury pattern	Repair
Baker et al. [8]	1980	Case 1	PT + ACL/MCL	Single stage (PT only)
Rae et al. [9]	1991	Case 1	PT + ACL/MCL	Single stage (PT/MCL only)
Levakos et al. [10]	1996	Case 1	PT + ACL/MCL	Single stage
		Case 2	PT + ACL	Two stage
		Case 3	PT + ACL	Single stage
		Case 4	PT + ACL/MCL	Single stage (PT/MCL only)
		Case 5	PT + ACL/MCL	Single stage (PT only)
		Case 6	PT + ACL/MCL	Two stage
Costa‐Paz et al. [11]	2005	Case 1	PT + ACL/MCL	Two stage
		Case 2	PT + ACL/MCL	Single stage
		Case 3	PT + ACL	Single stage
Chiang et al. [12]	2005	Case 1	PT + ACL	Single stage
Chow et al. [13]	2006	Case 1	PT + ACL	Single stage (PT only)
Futch et al. [14]	2007	Case 1	PT + ACL	Single stage
Shillington et al. [15]	2008	Case 1	PT + ACL/MCL	Two stage
Tsarouhas et al. [16]	2011	Case 1	PT + ACL/MCL	Two stage
Koukoulias et al. [17]	2011	Case 1	PT + ACL/MCL	Two stage
Wissman et al. [18]	2012	Case 1	PT + ACL	Single stage (ACL only)
Chiba et al. [19]	2013	Case 1	PT + ACL/MCL	Two stage
De Baere et al. [20]	2014	Case 1	PT + MCL	Single stage
Kim et al. [21]	2014	Case 1	PT + ACL/MCL	Single stage
Gulabi et al. [22]	2014	Case 1	PT + ACL/MCL	Single stage
Brunkhorst et al. [23]	2015	Case 1	PT + ACL/MCL	Two stage
		Case 2	PT + ACL/MCL	Single stage
Lobo et al. [24]	2017	Case 1	PT + ACL	Two stage
Cucchi et al. [25]	2018	Case 1	PT + ACL/MCL	Single stage
		Case 2	PT + ACL/MCL	Single stage
Quinn et al. [4]	2019	Case 1	PT + ACL/MCL	Two stage
		Case 2	PT + ACL/MCL	Two stage
		Case 3	PT + ACL/MCL	Two stage
Louka et al. [26]	2020	Case 1	PT + MCL (bilateral)	Single stage
Xie et al. [27]	2021	Case 1	PT + ACL/MCL	Single stage (PT/MCL only)
Scrivano et al. [28]	2022	Case 1	PT + ACL	Single stage
Current Report	2024	Case 1	PT + ACL/MCL/PCL	Two stage

Abbreviations: ACL, anterior cruciate ligament; MCL, medial collateral ligament; PT, patellar tendon.

The first case of this injury pattern was an ACL/MCL/PT injury in 1980. These authors treated only the PT rupture, leaving the ACL and MCL untouched as they were deemed to be irreparable [8]. In 1991, Rae and Davies reported a case of ACL/MCL/PT injury wherein they treated the PT rupture and the MCL tear, but left the ACL unrepaired [9]. In 1996, Levakos et al. reported a series of four ACL/MCL/PT injuries [10]. They performed three single‐stage reconstructions and one two‐stage reconstruction similar to that presented for our patient. This marked the first use of a staged reconstruction approach for this injury pattern, and the authors reported a positive outcome for the patient [10]. In the remaining ACL/MCL/PT cases reported between 1996 and 2022, some authors elected for a staged repair, whereas others attempted a single, simultaneous repair of all injured structures.

Conjecture still exists today over the best management of multiligament knee injuries, even without the confounding rupture of the PT. A recent Delphi study did provide expert consensus recommendations on the management of multiligament knee injuries including: (1) “that the current literature generally favors operative management of MLKI over nonoperative management,” (2) “that the decision to perform single or staged surgery should be made on an individual basis considering the pattern of injury (using an accepted classification system), associated injuries, patient factors, and the best available evidence,” (3) “that where possible, single‐stage surgery should be undertaken to facilitate early rehabilitation,” and (4) “that a decision to repair or reconstruct ligaments should be based on the severity of injury, tear location (proximal, midsubstance, and distal), and pattern of MLKI” [29]. One limitation acknowledged in the Delphi study is the data heterogeneity and predominance of lower order evidence that currently informs treatment decisions for this injury pattern [29].

Much of the literature available on this topic comprises systematic reviews and retrospective cohort studies with relatively small sample sizes. Many studies find no significant differences between single‐stage and two‐stage approaches for multiligamentous knee injuries. For example, a 2020 review from Ng et al. reported positive outcomes for both the single‐stage (acute) approach and the two‐stage approach when examining functional outcomes, failure rates of repair/reconstruction, and complications such as nerve injuries [30]. A 2018 systematic review identified no differences in range of motion, time to return to sport, or complication rates between the single‐stage and two‐stage approaches [31]. In 2022, a small study of 27 patients detected no clinical differences between multiligamentous injuries treated with the acute approach and those treated with the staged approach when examining functional outcomes by Lysholm and International Knee Documentation Committee (IKDC) scores [32].

However, a systematic review from Jiang et al. claims that a staged treatment approach yields the best clinical results for patients with ACL/MCL/PCL injury as evidenced by higher rates of patient‐reported “excellent” or “good” outcomes with the staged approach compared with the single‐stage approach [33]. Further, Mook et al. suggest that a staged approach may produce better subjective outcomes for the patient and result in fewer range of motion deficits as compared with the acute approach [34].

On the contrary, Lau et al. found that the single‐stage approach resulted in fewer complications, less reoperations, and lower total costs compared with the two‐stage treatment [35]. Again, the Delphi expert consensus recommends completing the single‐stage surgery wherever possible to facilitate early rehabilitation [29].

Regardless of repair technique, most authors agree that early postoperative rehabilitation is a critical factor influencing the patient′s final outcome [36]. Regarding rehabilitation protocol, the Delphi study expert consensus recommends “first, that a period of restricted weight‐bearing in a hinged knee brace between 4 and 6 weeks is preferred, but this is based predominantly on expert opinion; second, in the case of posterior cruciate ligament reconstruction (PCLR), the use of daily prone knee range of motion exercises with immediate quadriceps activation is advocated, rather than delaying mobilization.” [29] In this complex injury pattern, the PT and MCL injuries are repairable and can be fixed acutely, but the cruciate ligament injuries may require reconstruction in a second stage of surgery after the patient recovers from the acute PT/MCL repair.

## 4. Conclusion

To our knowledge, this is the first reported case of a combined tear of the ACL, PCL, and MCL ligaments with simultaneous rupture of the PT. Timely diagnosis of these injuries with the assistance of MRI is essential for developing an operative plan. In this complex case, a two‐staged operative repair was successful and provided positive clinical outcomes for the patient.

## Author Contributions

The authors confirm contribution to the manuscript as follows: **Nicholas I Chiaramonti**: writing – original draft, conceptualization, and investigation; **Nathan M Krebs**: writing – review and editing, methodology, and supervision.

## Funding

The authors received no specific funding for this work.

## Disclosure

All authors reviewed and approved the final version of the manuscript.

## Consent

The patient allowed personal data processing, and informed consent to publish was obtained from the patient in the study. Institutional review board approval was not required for this case report in accordance with institutional policy.

## Conflicts of Interest

The authors declare no conflicts of interest.

## Data Availability

The data that support the findings of this study are available on request from the corresponding author. The data are not publicly available due to privacy or ethical restrictions.
